# NIMH Project Accept (HPTN 043): Results from In-Depth Interviews with a Longitudinal Cohort of Community Members

**DOI:** 10.1371/journal.pone.0087091

**Published:** 2014-01-29

**Authors:** Suzanne Maman, Heidi van Rooyen, Petra Stankard, Alfred Chingono, Tshifhiwa Muravha, Jacob Ntogwisangu, Zipho Phakathi, Namtip Srirak, Stephen F.Morin, and the NIMH Project Accept (HPTN 043) study team

**Affiliations:** 1 Department of Health Behavior, University of North Carolina at Chapel Hill, Chapel Hill, North Carolina, United States of America; 2 Social, Behavioural and Biomedical Interventions Unit, Human Sciences Research Council, Pieternaritzburg, South Africa; 3 Department of Psychiatry, University of Zimbabwe. Harare, Zimbabwe; 4 Perinatal HIV Research Unit, Witswatersrand University, Soweto, South Africa; 5 Department of Psychiatry and Mental Health, Muhimbili University of Health and Allied Sciences, Dar es Salaam, Tanzania; 6 Research Institute for Health Sciences, Chiang Mai University, Chiang Mai, Thailand; 7 Center for AIDS Prevention Studies, University of California San Francisco, San Francisco, Califorinia, United States of America; Massachusetts General Hospital, United States of America

## Abstract

**Introduction:**

NIMH Project Accept (HPTN 043) is a community- randomized trial to test the safety and efficacy of a community-level intervention designed to increase testing and lower HIV incidence in Tanzania, Zimbabwe, South Africa and Thailand. The evaluation design included a longitudinal study with community members to assess attitudinal and behavioral changes in study outcomes including HIV testing norms, HIV-related discussions, and HIV-related stigma.

**Methods:**

A cohort of 657 individuals across all sites was selected to participate in a qualitative study that involved 4 interviews during the study period. Baseline and 30-month data were summarized according to each outcome, and a qualitative assessment of changes was made at the community level over time.

**Results:**

Members from intervention communities described fewer barriers and greater motivation for testing than those from comparison communities. HIV-related discussions in intervention communities were more grounded in personal testing experiences. A change in HIV-related stigma over time was most pronounced in Tanzania and Zimbabwe. Participants in the intervention communities from these two sites attributed community-level changes in attitudes to project specific activities.

**Discussion:**

The Project Accept intervention was associated with more favorable social norms regarding HIV testing, more personal content in HIV discussions in all study sites, and qualitative changes in HIV-related stigma in two of five sites.

## Introduction

One of the challenges of large-scale intervention trials is to adequately describe the contextual factors that influence study findings. Even in trials that demonstrate efficacy, the explanation of behavioral factors that influence the interpretation of results are enriched by a mixed-methods approach that incorporates social science methods, such as in-depth interviews, into the overall study design [1]. A mixed-methods approach is particularly important in the context of multi-level interventions that are designed to effect social and behavioral change at the community level, rather than the individual level alone. There are several examples in the literature of trials that have incorporated qualitative research into the study design [2]–[7]. Many of these studies included a qualitative, formative research component to inform the intervention and the quantitative assessment tools. There are fewer examples of trials that have included qualitative research in the evaluation design. Three notable examples in the literature embedded a longitudinal qualitative assessment among a sub-sample of study participants to document how the intervention influenced behavior, and in the case where null effects were found, to help understand why the intervention did not influence behavior as expected [2]–[4]. For example, in community-randomized trial of an intervention to promote adolescent sexual health in Tanzania, the qualitative findings suggested that one reason the intervention did not modify individual behavior is that the cultural belief systems relevant to adolescent sexuality did not change enough to support individual behavior change [3].

NIMH Project Accept (HPTN 043) is a clustered community-randomized trial to determine the safety and efficacy of a community-level behavioral intervention in reducing HIV incidence. The trial tests whether a community based model of HIV counseling and testing that incorporates community mobilization, increased access to testing and post-test support, can reduce HIV incidence at a population level rather than determining efficacy in changing behavior at the individual level. The parent study found significantly greater HIV testing in communities receiving the community-level intervention as compared to communities receiving the standard VCT. Almost four times more HIV cases were detected in the intervention communities than in comparison communities in three study sites (952 *vs* 264; p = 0·003) where a direct comparison was possible [8]. The quantitative assessment of secondary outcomes found that social norms regarding HIV testing were improved in intervention communities. The positive change in social norms was greater in men than in women, but the intervention effect was significant in both subgroups. The intervention also reduced risk among HIV-infected participants. The intervention did not affect the proportion of participants who reported having a conversation about HIV in the past six months, having experienced negative life events, or having disclosed their status to someone. HIV-related stigma, as measured by a stigma scale also did not change as a result of the intervention. Baseline mean stigma scores were low and dropped slightly in both arms in the post-intervention assessment [9].

The qualitative assessment is a key component of the Project Accept study design. We present the findings from in-depth interviews with members of the intervention and comparison communities conducted at baseline and 30 months after the intervention had launched. The purpose of this analysis was to describe, at the community level, the secondary outcomes, including social norms regarding HIV testing, HIV related discussions, HIV-related stigma, HIV risk behavior, and HIV-related negative life events at these two time points and contribute to our understanding of the intervention effect by describing how these outcomes changed over time.

## Methods

### Ethics Statement

All participants provided written informed consent to participate in this study. The study, including the informed consent procedures, was approved by ethical review committees for each site: The Johns Hopkins Bloomberg School of Public Health (Thailand and Tanzania), Chiang Mai University Research Institute for Health Sciences (Thailand) and Ministry of Public Health (Thailand); and Muhimbili University of Health and Allied Sciences (Tanzania), The Medical College of South Carolina (Tanzania), The National Institute of Medical Research (Tanzania), The University of California at Los Angeles South General Institutional Review Board (South Africa), The University of the Witwatersrand (South Africa), The University of California, San Francisco (Zimbabwe), and The Medical Research Council of Zimbabwe (Zimbabwe). All informed consent forms were translated and administered in the local language. Translated consent forms were reviewed and approved by ethical review boards that had oversight for each site. Interviews were conducted in or near the homes of participants. Interviewers found quiet spaces that would limit interruptions, allow for audio recording and insure privacy.

### Study Design

The qualitative assessment was embedded within the evaluation design for Project Accept (HPTN 043). Briefly, Project Accept was designed as a behavioral intervention with three major strategies: 1) Community Mobilization – designed to change community norms around HIV awareness, particularly the benefit of knowing one’s HIV status; 2) Increased Access to VCT– by removing barriers to knowing one’s HIV status and to reinforce the goal of making testing more normative, through provision of free, parallel rapid tests by mobile vans or in community settings with same day results. Testing was done in combination with condom distribution, individual risk reduction assessments, motivational interviewing and counseling to promote behavior change as well as linkage to available community services; and 3) Post-Test Support *Services* – designed to increase safety and minimize the potential negative consequences of testing by providing specific forms of support. The three strategies were designed to be synergistic and result in sustainable change in communities mediated by more adaptive community norms [10]. Project Accept (HPTN 043) was evaluated through a phase III clustered, community randomized trial in which 24 matched community pairs were randomized to receive a community-level intervention or serve as comparison communities. The study protocol is available online at http://www.cbvct.med.ucla.edu/protocol.pdf. Participants in the qualitative assessment were interviewed four times throughout the study period at baseline, 6, 15 and 30 months of intervention. With the primary goal of describing patterns change at the community level in the secondary endpoints from baseline to the end of the intervention period, the data for this analysis were drawn from the baseline and 30-month interviews conducted between January, 2005 and June, 2009. This article extends the analysis of the baseline qualitative data described by Maman et al. [11].

### Study Sites

The trial was conducted in four countries, including 10 communities in Kisarawe, Tanzania, 8 in Mutoko, Zimbabwe, 16 in South Africa (8 in Vulindlela, KwaZulu-Natal, and 8 in Soweto, Gauteng) and 14 in Chiang Mai, Thailand. The study sites were all rural with the exception of Soweto, South Africa, which is a densely populated urban area of Johannesburg with just over 1 million inhabitants. The second site in South Africa, Vulindlela, is a sub-district within the KwaZulu-Natal midlands region. It is situated about 140 km. from Durban, and has a total population of approximately 400,000. In Tanzania, the study site is located in Kisarawe, a rural district of approximately 100,000 people located 30 km. northwest of Dar es Salaam. The Zimbabwe site is located in Mutoko, a rural district, with approximately 130,000 residents, located 150 km. from Harare. The only Asian site included in the trial is located within Chiang Mai Province, in Northern Thailand. The study communities are located in a mountainous area between 40–135 km. from Chiang Mai City. At baseline there were site-specific differences in terms of access to HIV and other health services. At the outset, South Africa had a sizeable HIV/AIDS budget, with 31% coverage of antiretroviral therapy (ART) and with VCT services being increasingly rolled out across communities. In contrast, Zimbabwe and Tanzania had modest HIV budgets with ART coverage in the region of 15–18%, and virtually no HIV voluntary counseling and testing services in study communities [12]–[14].

### Study Participants

**Figure 1:**
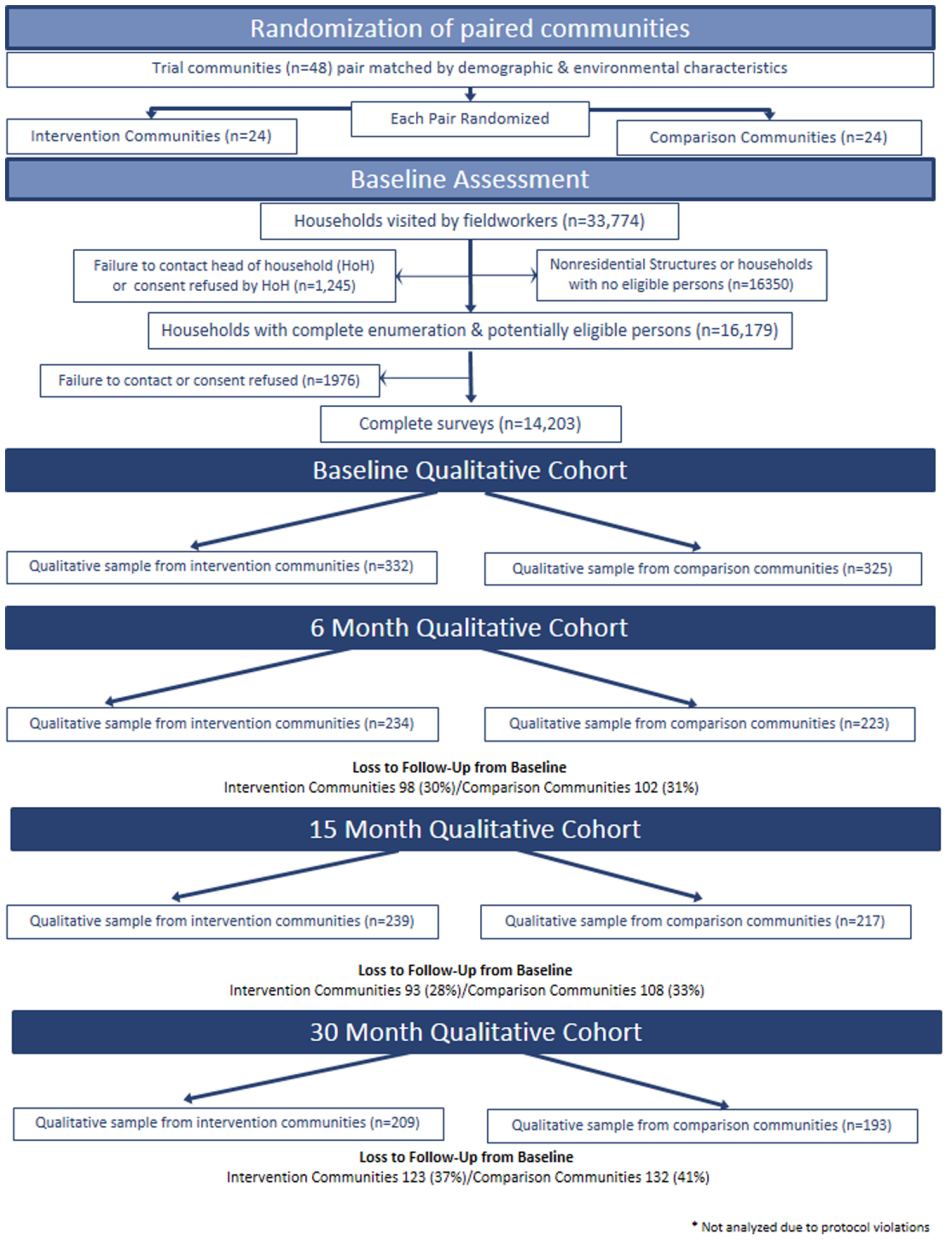
Flow of participants from baseline assessment through 30-month follow-up.

Participants in the qualitative assessment were community members who were recruited from individuals who participated in the baseline behavioral survey conducted for the main trial. The baseline and post-intervention survey were conducted among random samples of community residents 18–32 years of age regardless of their participation in intervention activities. Participants for the survey were selected for outcome assessment by two-stage random sampling from the population of eligible community residents. At the first stage, households were selected with equal probability from a listing of all households prepared by the study staff. At the second stage, detailed behavioral outcomes were assessed on a single randomly selected household member. The baseline assessment included 14,657 participants across the five sites, and 56,683 participants completed the post-intervention behavioral assessment [9]. The qualitative cohort was stratified into eight demographic categories according to gender, age (18–24 years and 25–32 years), and partner status (single or coupled). The list of baseline survey participants were divided into the three demographic categories. We randomly selected two participants per category in each of the African communities and one participant per category in each of the Thai communities because we had more Thai communities in the trial. Due to the size of this trial, and our goal to document and compare community-level change over time from the perspective of community members in these different categories, this sampling strategy resulted in a qualitative sample that sought to balance representation with feasibility. Sampling yielded a total of 657 individuals in the baseline in-depth interviews across all sites. In anticipation of loss to follow-up, we oversampled cohort participants in every category, ultimately retaining a total of 402 participants throughout the 30-month follow up period. See Figure 1 for a description of the flow of participants from the baseline behavioral survey through the qualitative assessment.

### Data Collection and Analysis

The in-depth interviews were semi-structured based on a standard field guide that was used across all sites. We trained teams of between 5–9 local interviewers in qualitative research methods using a standardized training manual implemented across the five sites. Training focused on building qualitative data collection skills including asking open ended questions, probing, and active listening through the use of demonstrations, role plays and practice interviews. A knowledge and skills assessment was conducted at the end of the training to insure that each interviewer was prepared to conduct the interviews. Interviewers were part of the assessment team for each site, and did not have any intervention implementation responsibilities. Interviewers conducted 30–60 minute in-depth interviews in the local languages that included Kiswahili in Tanzania, IsiZulu in Vulindlela, Shona in Zimbabwe, English, isizulu, Sesotho, Sepedi, Setswana, Tshivenda, and Xitsonga in Soweto, and Thai and Lau in Thailand. Only one interview per time period was conducted with each informant. Interviewers reviewed data from previous waves of data collection for each informant prior to conducting interviews to insure that they followed up on ideas raised by the informants in each wave.

The qualitative cohort explored the following attitudinal and behavioral study outcomes: HIV testing norms, HIV related discussions, HIV related stigma, HIV risk behavior, and HIV-related negative life events. We discerned no apparent changes in risk behavior among either HIV-negative or HIV-positive study participants. There were insufficient numbers of qualitative cohort members who self-identified as being HIV positive (12 in Vulindlela, 10 in Soweto, 6 in Zimbabwe, 1 in Thailand and none in Tanzania) and who could report on HIV-related negative life events across study arms, so we neither attempted this analysis nor report the results here. Therefore, this paper will focus on HIV testing norms, HIV related discussions and HIV related stigma. Table 1 presents the questions that were included on the interview guides to explore these three outcomes.

**Table 1:** Questions included on the interview guide to explore outcomes.

Outcome	Questions that were asked
HIV testing norms	How would someone in this community learn their HIV status? Are you aware of a test you can take to learn your HIV status? Do you ever talk to people about tests that can be done to learn your HIV? Do you know anyone in this community who has taken this test? If you have tested for HIV can you tell me about your most recent testing experience? Can you tell me about any discussions you may have had before you were tested? You mentioned you have not tested for HIV, can you tell me about your decision not to test for HIV?
HIV-related discussions	Can you tell me about the last conversation you had with someone when you talked about HIV? Do you ever talk to anyone about tests that can be done to learn your HIV status? Can you tell me about any conversations you have had with your partner about HIV and your risk for HIV?
HIV-related stigma	How do people in this community feel about HIV? Can you tell me about anyone in your community who has been affected by HIV? If someone you know told you that they were infected with HIV, how would you react to them? How do you feel about this person?

We had several levels of staff involved in the analysis. At each site there was a Qualitative Supervisor who trained and supervised the data collectors, data processors and data analysts at their site. All interviews were audiotaped, transcribed and translated into English for analysis. Qualitative Supervisors checked the transcription and translation quality by reviewing 10% of the interview transcripts against the original audio files. Following transcription and translation, the data were indexed by topics through the application of topical codes by teams of 3–5 local data coders [15]. The codebooks were organized by topics that were included on the interview guide. There were six main topics and 32 sub-codes within these topics on the codebook. The codebook included a definition of the code and instructions about when to apply and when not to apply each code. The training manual for the codebook included examples of text that could be coded with each of the codes included in the codebook. We developed standardized workshops to train these staff in the codebook, the application of codes, and the use of Atlas.ti. To maintain coding quality each coder completed a certification exercise prior to initiating the work. Qualitative Supervisors checked 10% of all coded transcripts, with more transcripts being checked early in the coding process to catch errors early. The senior data analysts spot checked the quality of the coding from each site during each wave of data collection, by reviewing at least 5 selected transcripts per site per wave. The coding team worked with the interviewing team at each site to clarify questions they had when reviewing and coding data and to share impressions of the data as they made progress. A team of senior data analysts were responsible for cross-site analysis of the data. The senior analysts were assisted by a team of five analysts who were trained in social and behavioral sciences at the Master-level (4) and doctoral level (1). They were responsible for generating summary reports for each site. The analysts used the data that was topically coded to generate code reports for each of the study outcomes. They wrote detailed summary reports for each main code and sub-code that noted insights, key themes, illustrative quotes, and patterns that emerged in the data related to each topic at baseline and at 30-months. The summary reports for each site at each wave was on average 175 single spaced pages. The team of senior data analysts and data analysts had regular conference calls during the process of creating the summary reports to share progress and compare and discuss findings within each section. Identifiers of intervention/comparison communities were removed from the transcripts prior to the review of 30-month data in order to blind analysts to randomization status. The analysts, reflected on the data summaries from each wave, and then created a separate document that described any changes that they noted over time in the topics. Where no changes were observed, this was also noted. Once the general patterns of change had been described, the code reports were separated by intervention and comparison communities to determine if the patterns of change were different for these different communities. We analyzed the data in a cross sectional way by wave because our focus for this analysis was on describing and understanding change at the community level over time, not on describing individual level processes of change. A total of 1059 in-depth interviews were analyzed, including 657 baseline interviews and 402, 30-month interviews.

## Results

### HIV Testing Norms

While the perceived availability of HIV testing increased among participants in all study communities over time, participants from the intervention communities were much more specific about where they could get tested, and what the benefits of testing were at 30-months.

Participants in comparison communities in all sites identified more HIV testing sites at 30-months, however, they were less likely to describe them as easily available, as this Zimbabwean participant describes:

*I: How would someone in your community learn their HIV status if they wanted to know?*

*P: They would have to go for tests at the hospital.*

*I: So are there many HIV testing centers available in this community?*

*P: No, we only have one center at Chindenga which opened recently, this year, the rest of us would want to take tests but these services are not readily available.” (Single female, older than 25 years)*

In contrast at 30-months, participants from intervention communities in Zimbabwe, Tanzania and Thailand described detailed awareness of testing sites due to Project Accept activities specifically, as expressed by this participant in Tanzania:

*I just see AFIKI (local name of the Project Accept) in the Tanzania site) because when they come they build tents and then people go to test…At AFIKI many people were going to test.” (Single female, older than 25 years)*

Different patterns in how testing was described also emerged. Participants from intervention communities in Thailand, Zimbabwe, Vulindlela and Tanzania talked in more detail about other benefits of testing, beyond just ‘knowing one’s status.’ Participants in these intervention communities referred to the preventive and other health benefits of testing, and mentioned being motivated to test by a community member they knew who had already tested, as expressed by this participant from Vulindlela in a 30-month interview:

*I remember one of the days I took the guys that I am working with….We took a walk to town, we went for testing….We were three and we convinced each other. (Coupled Male, less than 25 years)*

Unlike other sites, among Zimbabwean participants in intervention communities, treatment access emerged as a key theme motivating testing. Whereas the benefits of testing described by participants in the comparison sites at 30-months remained general as described by this older, coupled female from Zimbabwe, *“I motivate myself. I want to be tested so that I know where I stand. That will help me.”.*

Barriers to testing that were mentioned at baseline included logistical barriers related to distance/access, and distrust in the testing process. Some of these same barriers emerged again in the data at 30-months but less frequently overall and notably less often among participants from intervention communities. Logistical barriers including distance and cost dropped off almost completely among participants from the intervention communities in all sites.

### HIV-related Discussions

Over time, the context of HIV-related discussions shifted to a focus on HIV testing in both intervention and comparison communities. At baseline in the African sites, when asked about HIV-related discussions with their partners, participants made little to no mention of HIV testing. By 30-months, testing was one of the most common and important points of discussion that people mentioned as illustrated in the quote below from Zimbabwe:

*“I: Can you tell me again about the discussion on HIV you might have had in your family, with a friend or someone from the community?*

*P: Yes, I had the discussion with my husband because at first he did not want to get tested but I was going for testing alone and then I later said no he should go for testing as well and he said that if he gets tested and is found to be HIV positive he will commit suicide. Then I said a person should not commit suicide but it is good for one to know their status so that they live a better life because even if you want to have a baby you will know your status and know how you can protect yourself. That is how he got to accept it and then went for testing.” (Coupled Female, less than 25 years)*

In Zimbabwe and Vulindlela, discussions about testing dominated HIV-related discussions with partners, family and friends at 30-months and overshadowed mention of other risk reduction strategies including encouraging partners to remain faithful, and using condoms. In Thailand, individuals in both intervention and comparison communities described a community-level decline in HIV-related discussions because the perception of HIV as a problem decreased.

The nature of these HIV testing discussions were different among participants in intervention and comparison communities. At 30-months intervention community participants in all sites talked about testing in more detailed and personal ways, often referring to their own testing experience. There were also fewer negative references to HIV testing in intervention communities at 30-months. In Zimbabwe, for example, individuals in the comparison communities talked very simply about testing to “know one’s status” at baseline and at 30-months. A typical description from a comparison community participant went as follows:

*“I: What do you mostly discuss?*

*P: That it’s good to take HIV tests so that you will be aware of your status.*

*I: What are the benefits of knowing your status? Are there any benefits?*

*P: The benefits are that you would get to know of your status and can probably take steps to prolong your life.” (Single Female, less than 25 years)*

Whereas at 30-months individuals in the intervention communities talked about the importance of testing in more detailed ways such as for accessing treatment as described by this informant from Zimbabwe:

*“Yes I had with my niece who I advised get tested because these days treatment is based on one having a blood test…Like these days if you go the clinic they will first ask for the card which shows that your blood was tested. If your blood has not tested they will say “we are not treating. We can’t treat you of your disease if we do not know it” (Coupled Female, older than 25 years)*

In Tanzania and Zimbabwe, there was a clear pattern in which testing discussions among community members in the intervention sites were often grounded in their own testing experiences. Participants talked about discussions they had had with their partner prior to testing and after they had been tested together.

*“Because I had a partner who is the father of my child but he later died. So when I got another man, I told him that if he wants to be with me then we have to go and get tested. I am thankful that he agreed. Went together and yes and we got our results together.” (Single Female, less than 25 years, Tanzania)*

Individuals in the African sites made direct reference to the availability of HIV testing and treatment as a factor that led them or someone they know to get tested. In Zimbabwe and Tanzania access to VCT was directly attributable to Project Accept, and differences in HIV-related discussions could be differentiated by intervention and comparison communities at 30-months. Participants in these two sites referred to the HIV “education” they received during the community mobilization activities that prompted them to talk more about HIV testing with their partners, family and friends. In Thailand, participants from the intervention communities made many references to Project Accept activities and acknowledged that these intervention initiatives resulted in more discussion about HIV testing in their communities. In the two South African sites, while participants also attributed changes in HIV-related discussions to increased access to testing and treatment, other efforts to expand testing in these communities outside of Project Accept made it difficult to attribute changes specifically to intervention initiatives.

### HIV-related Stigma

We noted a decrease in stigmatizing language and attitudes in all of the African sites over time. However, at baseline the stigmatizing attitudes and behaviors were more pronounced in the Zimbabwe and Tanzania sites. There was fear associated with casual contact and with the perceived inevitable death associated with HIV in these two sites. There was also a greater blame associated with HIV among participants in these two sites and greater sense of resentment about the burden that PLWHA place on family. These feelings of blame and burden are expressed by this participant at baseline from Zimbabwe:

*“Right now those who are infected are not treated as fellow human beings. They are already declared dead, and regarded as useless as a grave. That is how they are treated…They mean that these people are no longer able to do anything useful. They say they are just waiting for the day of their death.”(Younger single male, Mutoko, Zimbabwe)*

While there was a growing awareness of HIV treatment in all sites, and this led people to think about living a longer and healthier life with HIV, the perceived benefits of treatment seemed to have the greatest effect on attitudes towards PLWHA in the Zimbabwe and Tanzania intervention communities by 30-months. As a result of not manifesting physical symptoms of the virus, individuals shifted their description of people living with HIV/AIDS away from characterization of them as threats or burdens in their communities as described by this participant from Zimbabwe:

*Yes. There are changes I have observed. There were some people who were not satisfied that AIDS is… a person who has AIDS can lead a life which is just the same as the one for someone who is negative because people used to have the idea that once a person is found with the virus then they die. There are people who were diagnosed with the HIV many years ago… about 1997 until now we could say for 10 years but they are still alive. They are still strong and are doing work just like everybody. So this is a sign to people that if you are diagnosed with the virus, it is a disease which is just like other diseases, it does not mean that you are faced with death but that you could change your life style and live a new life style.”(Coupled male, older than 25, Mutoko, Zimbabwe)*

In the Zimbabwe and Tanzania intervention communities, the change in attitudes was attributed specifically to education that participants had received through Project Accept in addition to the effect of increased treatment availability. This education led to a change in the themes that emerged around HIV transmission–away from concern about transmission through casual contact, and toward an understanding that anyone could get infected with HIV, as described by this participant from Tanzania,

*“In the past they used to see him/her differently. They used to say, ‘Daah, that person is infected.’ But nowadays they take it as a normal thing. And this is because they have been educated about the issue… It is because this disease does not choose, so today it can be his problem but tomorrow it can happen to you as well.” (Coupled Male, less than 25 years)*

## Discussion

These qualitative data were collected to enhance our understanding of whether and how the intervention effected changes in the study communities. In the discussion that follows we compare and discuss the qualitative and quantitative assessment of study outcomes relating to HIV testing norms, HIV related discussions, HIV risk behaviors and HIV related stigma.

With regard to HIV testing norms, in the qualitative assessment we found more favorable community norms regarding HIV testing in the intervention communities, which is similar to what was found through the assessment of quantitative behavioral outcomes. In both cases, individuals in intervention communities reported fewer barriers and more benefits to testing. Our qualitative data enhanced the understanding of this intervention effect by describing how the members of the intervention community were more specific, detailed and personal in their descriptions of the benefits of testing.

The quantitative data did not support the hypothesis that Project Accept would lead to more frequent discussions about HIV. Our in-depth interviews shed light on differences between intervention and comparison communities, not in terms of frequency of the discussions but in terms of the nature and content of these discussions. Specifically, we found that over time HIV-related discussions became dominated by HIV testing in all communities, and the discussions in intervention communities were more detailed and more often grounded in personal testing experiences.

Reducing HIV-related stigma was a primary goal of the Project Accept intervention. Our survey data comparing baseline to post-intervention levels of stigma using a stigma scale failed to support the hypothesis of an intervention effect [16]. These quantitative findings are consistent with findings from other trials of HIV testing. Trials of home-based testing from Zambia and South Africa found that this approach did not lead to a greater reduction in stigma. In a trial from Kenya, home-based testing led to decreased stigma among community leaders but did not lead to a decrease in stigma at the community level in the intervention communities [17]–[19]. The data from our in-depth interviews present a different picture. We found more widespread and detailed accounts of stigma in our cohort at baseline than what was found through the baseline behavioral survey, particularly in Tanzania and Zimbabwe [11], [16]. In our baseline qualitative paper on stigma, we suggested that the differences we saw in stigma across the project sites could be understood in light of the severity of the epidemic, and the amount of care and treatment resources that were available to respond to the epidemic in each site [11]. The HIV epidemics in Tanzania and Zimbabwe are high, and at baseline there were very few resources that were available in the project communities to provide PLWHA. Treatment and care resources to respond to the epidemic were and have remained low relative to the other sites. In our qualitative analysis, we noted a change in the pattern of attitudes related to stigma in these two sites, which may be due to the fact that Project Accept offered services in settings where there were little to no other services available. While ART access increased in all sites, the larger number of other HIV testing and treatment initiatives in South Africa made it difficult to attribute changes to Project Accept specifically. While treatment access was increasing in Tanzania and Zimbabwe as well, there were fewer other initiatives happening in conjunction with Project Accept and thus the effect of the intervention on attitudes and beliefs was clearer in these sites. There have been findings from other studies to show that there is not a direct correlation between greater access to ART and reduced stigma. In fact, some studies have found that ART roll-out has led to new forms of stigma, and have increased community concerns that as PLWHA become physically healthier traditional methods of assessing risk by looking at the physical condition of people can no longer be used, and therefore there is fear that PLWHA will hide their conditions and intentionally put people at risk for HIV by engaging in unprotected sex [20]–[22]. At the time of these qualitative interviews, the reality of ART roll out in the communities within Tanzania and Zimbabwe where the study was conducted was still limited. So while there was greater awareness about ART, there was still limited personal experience with knowing PLWHA on ART. It is likely that the stigmatizing attitudes that develop as a result of ART, may develop over time as individuals have more personal experiences with ART. Data collected from Thailand were quite different in their themes from the African sites reflecting the low HIV prevalence and less urgency about the epidemic in that context.

Finally, we had included sexual risk behavior in our analysis but did not identify any meaningful patterns of change over time and have not presented those data in this paper. The quantitative paper on secondary study outcomes found a significant reduction in sexual risk behavior over time among HIV-infected community members only [9]. We did not have a sufficient number of HIV-infected participants in the in-depth interviews to assess behavior change in this group. Also, as described above, we did not attempt to analyze patterns in HIV-related negative life events because of the small number of individuals who self-reported as being HIV infected through the qualitative interviews.

This study is not without limitations. We are reporting on qualitative data which by its nature is better at describing contextual issues than frequencies, which is how the behavioral and attitudinal hypotheses for this trial were framed. Rather than using a probability based sample, we designed the in-depth interview cohort to reflect key demographic categories that we believed would influence the behavioral and attitudinal outcomes. While our sample was large for a qualitative study, the sampling and the methods were not designed to detect statistically significant intervention effects [23]. Yet, in an intervention trial of this scale, the qualitative sample had to be large enough to describe and compare the processes of community-level change over time in all the different sites. The strength of our sampling approach is that it provided us with a sufficiently large and diverse sample at baseline and in the post-intervention period to be able to describe patterns of community-level change. The multi-site design of data collection constrained us in terms of how flexible we could be at each site with regard to exploring emerging themes. We needed comparable data across the sites to conduct this evaluation, thus we opted for structure versus flexibility. While the topics for the interviews were pre-determined, the strength of these methods is that they allowed for different patterns to emerge within and across the different sites for each topic. In addition, due to the multi-site nature of the study the data were collected in multiple languages, which then had to be translated into English for analysis. Inevitably some quality of the narratives was lost in the translation process [24]. Through repeated opportunities to talk with interviewers, the participants may have identified what they felt was the correct response to questions related to the attitudes and behaviors. Through these methods we asked participants to narrate in detail their experiences, making it more difficult for people to easily mask what may have been their attitudes or behaviors in favor of what they believed to be a correct response. However, it is still possible that individuals may have been primed to answer in a socially desirable way by repeated exposure to the questions. We chose not to include the data from the 6- and 15-month waves of data collection in this analysis because we were focused on characterizing broad changes over time that may have been attributed to the intervention, and because we wanted our qualitative analysis to parallel the analysis of quantitative secondary outcomes. Inclusion of the data from the 6- and 15-month waves of data collection would have allowed us to document more incremental changes over time, which could be valuable in terms of understanding how the communities changed in response to the intervention. We characterized community level attitudes by asking a collection of individuals about perceptions, and by aggregating the individual responses from all individuals. It may have bolstered our understanding of community-level changes if we had collected community-level data to characterize these changes. Irrespective of these limitations, we believe that the incorporation of qualitative research in the evaluation design provided us with an opportunity to understand how and why changes happened in this intervention trial.

## Conclusion

The qualitative research was an integral part of the evaluation design of NIMH Project Accept, a community-randomized trial of community-based HIV counselling and testing. The qualitative findings describe how HIV testing norms and HIV related discussions changed over time in the intervention communities as opposed to matched comparison communities. Moreover, in the two study sites with the most limited HIV-related resources, we were able identify changes in HIV-related stigma in the intervention communities that were not readily detected in the other three sites, or in the survey data. Overall, the Project Accept intervention has led to increased detection of HIV, a decrease in HIV risk behavior among HIV-infected community members as well as a number of important changes in attitudes and behaviours at a community level. Lessons learned from this study will be important as countries with significant HIV burden design and implement prevention and care programs.
